# Neuroprotective role of nitric oxide inhalation and nitrite in a Neonatal Rat Model of Hypoxic-Ischemic Injury

**DOI:** 10.1371/journal.pone.0268282

**Published:** 2022-05-11

**Authors:** Peter Jung, Euntaik Ha, Meijuan Zhang, Carolyn Fall, Mindy Hwang, Emily Taylor, Samuel Stetkevich, Aditi Bhanot, Christopher G. Wilson, Johnny D. Figueroa, Andre Obenaus, Shannon Bragg, Beatriz Tone, Saburi Eliamani, Barbara Holshouser, Arlin B. Blood, Taiming Liu

**Affiliations:** 1 Division of Neonatology, Department of Pediatrics, Loma Linda University School of Medicine, Loma Linda, CA, United States of America; 2 Center for Perinatal Biology, Loma Linda University School of Medicine, Loma Linda, CA, United States of America; 3 Center for Health Disparities and Molecular Medicine, Loma Linda University School of Medicine, Loma Linda, CA, United States of America; 4 Department of Pediatrics, School of Medicine, University of California, Irvine, CA, United States of America; 5 Center for Imaging Research, Department of Radiology, Loma Linda University School of Medicine, Loma Linda, CA, United States of America; Universita degli Studi di Napoli Federico II, ITALY

## Abstract

**Background:**

There is evidence from various models of hypoxic-ischemic injury (HII) that nitric oxide (NO) is protective. We hypothesized that either inhaled NO (iNO) or nitrite would alleviate brain injury in neonatal HII via modulation of mitochondrial function.

**Methods:**

We tested the effects of iNO and nitrite on the Rice-Vannucci model of HII in 7-day-old rats. Brain mitochondria were isolated for flow cytometry, aconitase activity, electron paramagnetic resonance, and Seahorse assays.

**Results:**

Pretreatment of pups with iNO decreased survival in the Rice-Vannucci model of HII, while iNO administered post-insult did not. MRI analysis demonstrated that pre-HII iNO at 40 ppm and post-HII iNO at 20 ppm decreased the brain lesion sizes from 6.3±1.3% to 1.0±0.4% and 1.8±0.8%, respectively. Intraperitoneal nitrite at 0.165 μg/g improved neurobehavioral performance but was harmful at higher doses and had no effect on brain infarct size. NO reacted with complex IV at the heme *a*_3_ site, decreased the oxidative stress of mitochondria challenged with anoxia and reoxygenation, and suppressed mitochondrial oxygen respiration.

**Conclusions:**

This study suggests that iNO administered following neonatal HII may be neuroprotective, possibly via its modulation of mitochondrial function.

## Introduction

Neonatal encephalopathy is a leading cause of morbidity and mortality in neonates, affecting 2 to 5 newborns per 1000 live births [1–3]. Neonatal encephalopathy is initiated by a severe asphyxic episode due to inadequate uterine or umbilical blood flow before birth, or prolonged apnea shortly after birth, leading to an acute decrease in systemic oxygen delivery to the fetus or infant. The hypoxic-ischemic insult (HII) results in deprivation of cerebral oxygen and glucose supply, which causes primary energy failure and initiates a series of secondary injurious events leading to cell dysfunction and, ultimately, to cell death [4]. Mitochondria have been proposed to be the central hub that determines the fate of brain cells subjected to HII [5, 6].

Nitric oxide (NO) is a freely diffusive gas naturally produced in the body. Due to its short half-life, milliseconds within blood [7], NO was conventionally thought to be active only locally, with no endocrine effects. However, this has been challenged by observations that NO metabolites (NOx), such as nitrite [8], actually preserve NO-like bioactivity that can circulate in blood. Studies have shown that iNO administration leads to an increase of nitrite in plasma and cerebrospinal fluid nitrite concentrations [9, 10]. Although the mechanisms underlying the NO-like effects of nitrite remain unclear, a commonly proposed theory is that it is converted back into NO via reactions that are favored by conditions of acidosis, hypoxia, and ischemia [11, 12]. NO could be cytoprotective in a variety of ways, such as vasodilation to support O_2_ delivery, modulation of apoptosis, and suppression of excitotoxicity. In addition, NO may modulate mitochondrial function by interacting with complex I and IV, resulting in suppression of O_2_ consumption and reactive oxygen species (ROS) production, making it a potential mitochondria-targeted therapy [13–15].

Numerous studies have investigated the effects of iNO in various animal models of neurologic injury. It has been shown to be protective in neonatal hyperoxic (rats) [16], neonatal excitotoxic (rats) [17], and adult traumatic (mice) brain injury [18]. However, studies of HII have produced mixed results. Examples include findings of protection in rats [19], protection in male [20] but not female mice [21], and harm in rats [22]. Akin to iNO, nitrite has also been shown to be protective in a broad range of animal models and organ systems against ischemia-reperfusion injury, including cardiac arrest (mice) [23], stroke (rats) [24], and subarachnoid hemorrhage (baboons) [25]. To date, no studies of the neuroprotective effects of nitrite have been conducted in newborn animal models.

Our objective was to evaluate the effects of exogenous NO given as iNO and sodium nitrite on the Rice-Vannucci model of HII in neonatal rats. We hypothesized that NO is neuroprotective via its modulation of mitochondrial function. To test this hypothesis, we examined the effects of iNO and nitrite on brain infarct sizes, inflammatory cytokine levels, and neurobehavioral outcomes. We also examined the reaction between NO and isolated brain mitochondria and measured its effect on mitochondrial function.

## Methods

All procedures and protocols involving animals were preapproved by the Institutional Animal Care and Use Committee of Loma Linda University (Approval#: 8160019) and followed guidelines of the National Institutes of Health Guide for the Care and Use of Laboratory Animals.

### Neonatal Rat Model of Hypoxic-Ischemic Injury

A modified Rice-Vannucci model [26] was used to induce HII. Briefly, postnatal 7-day-old (P7) Sprague-Dawley rat pups (Charles River Laboratories, Portage, MI, USA) of mixed males and females were fully anesthetized with inhalation of isoflurane (4% for induction and 2% for maintenance; total duration of 5 to 10 min). The right common carotid artery was isolated, double-ligated with an 8.0 silk surgical suture, and then severed between the two ligation sites. After surgery, pups were recuperated at 37°C for approximately 15 minutes and then returned to the dam. The entire recovery period lasted between 1.5 to 2 h. Then, the pups were placed in a hypoxic incubator containing humidified 8% O_2_ balanced with 92% N_2_ for 1.5 h at 37°C. After the hypoxia period, pups were returned to their dams for recovery. Animals in the sham group were treated similarly, except the right common carotid artery was surgically exposed but not ligated and they were not exposed to hypoxia.

### iNO administration

**Fig 1:**
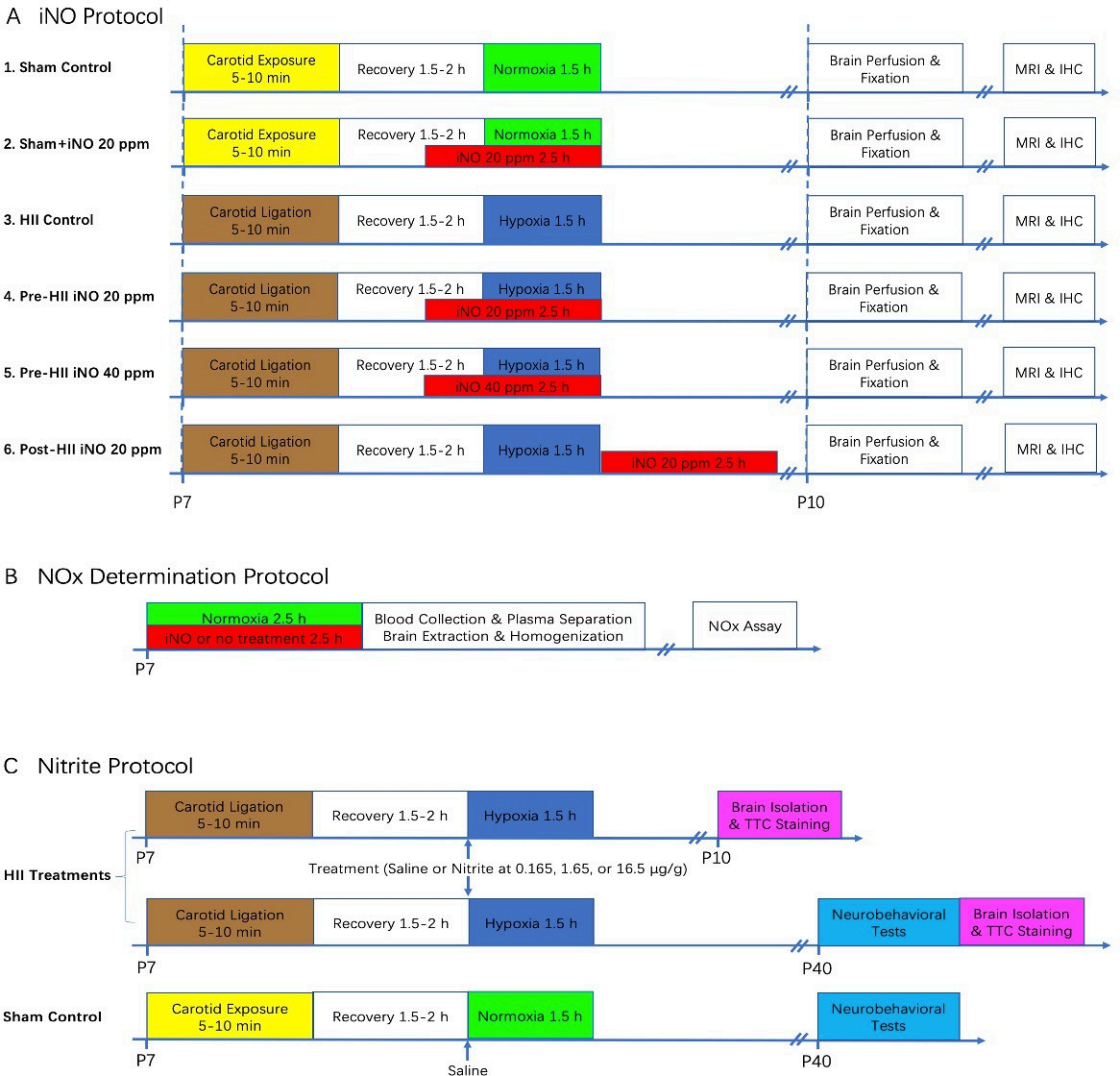
Experimental protocols for three neonatal rat studies. **A)** Tests for effects of iNO on HII. **B)** Measurements of NOx in plasma and brain tissue of animals that received iNO. **C)** Tests for effects of nitrite on HII.

Rat pups were randomly assigned to one of six experimental groups according to the iNO protocol shown in Fig 1A. Inhaled NO was administered using an INOmax DS_IR_ Plus delivery system provided by Mallinckrodt Pharmaceuticals (Bedminster, NJ. USA). To test for the effects of iNO given before or after the HI insult, the 2.5 h iNO (20 or 40 parts per million (ppm)) was started 1 h prior to (pre-HII) or immediately after (post-HII) the 1.5 h hypoxic period, respectively.

### NOx determination

Rat pups were assigned to one of three groups, and treated according to the NOx determination protocol (Fig 1B). One group received no treatment, the other two received iNO for 2.5h at 20 or 40 ppm, respectively. All pups were then sacrificed for collection of blood and brain tissue. Samples were snap frozen and stored at -80°C until analysis with an ozone-based chemiluminescence NO analyzer (Sievers NOA-280i) using tri-iodide as reagent. This analysis measures the total concentration of NO metabolites including nitrite, S-nitrosothiol (SNO) [27], and dinitrosyl iron complexes (DNIC) [28], and heme-NO complexes, but not nitrate [29].

### Nitrite administration

Rat pups were assigned to one of three groups and treated according to the nitrite protocol (Fig 1C). One group received saline treatment but not HII as the Sham Control, while the other two (HII groups) received HII and treatment with either saline (HII Control) or nitrite (HII Nitrite). Neurobehavioral tests were performed on all three groups at P40. To test for short- and long-term brain injury, additional pups in both HII groups were sacrificed for TTC staining at P10 (72 h post HII) and at P40 (after the neurobehavioral tests), respectively. Sodium nitrite (Sigma-Aldrich, St. Louis, MO, USA) was given at the end of the recovery period by i.p. injection (50 μl) at 0.165, 1.65, and 16.5 μg/g as the low, middle, and high doses, respectively.

### Magnetic resonance imaging (MRI)

The brain samples were suspended in Fluorinert for imaging with Bruker 11.7-T BioSpec MRI (Billerira, MA, USA) utilizing diffusion weighted imaging (DWI). MRI images were extracted as 0.5 mm thick coronal sections, resulting in about 20 slices for full brain coverage. Image analysis was carried out using ImageJ (bundled with Java 1.8.0_172) by an investigator who was blinded to the pup group assignment. The whole brain volume and total infarct volume were calculated from the sum of the area measurements across all slices, and infarct volume was normalized to brain volume.

### Inmmunohistochemistry (IHC) Staining

MRI brain samples were incubated with 30% sucrose and extracted as 20 μm coronal cryostat slices for IHC study using a brightfield staining protocol. Inflammatory responses to HII in the right hippocampal region, including glial fibrillary acidic protein (GFAP), ionized calcium binding adaptor molecule (IBA1), and interleukin-6 (IL-6), were analyzed by investigators blinded to the treatment groups using unbiased stereology software (SRC Biosciences; Tampa, FL, USA).

### TTC Staining

Lesion size calculated as the percent of total brain was determined as previously described [30].

### Neurobehavioral tests

CatWalk XT® (Noldus Information Technology, Wageningen, Netherlands), a quantitative gait analysis system, was used to assess motor and coordination performance as previously described [30]. The details are provided in S1 File.

### Mitochondria isolation

In preparation for future work in a sheep model of HII, mitochondria were isolated from the near-term fetal sheep brain cortex using a modified Percoll density gradient centrifugation methodology [31, 32]. The details are given in S1 File.

### Electron Paramagnetic Resonance (EPR)

EPR evidence for the reaction (37°C, 30 min) of NO (100 μM) with mitochondria (0.4 μg/μl) was recorded at 100 K using a Bruker X-Band EMX Plus EPR as previously described [28].

### Aconitase activity assay

Mitochondria (0.4 μg/μl) in assay buffer containing 4 mM ADP were treated with 30 μM NO or NO metabolites nitrite, glutathione-SNO (GSNO), and binuclear DNIC (BDNIC) at 37°C under normoxia or anoxia for 40 min. Then, the samples were centrifuged and resuspended in an oxygenated assay buffer containing 4 mM ADP followed by 20 min of incubation. The mitochondria were lysed by three freeze-thaw cycles for assay of aconitase activity with a commercially available kit (K716; BioVision, Inc.; Milpitas, CA, USA) [14]. GSNO and BDNIC were synthesized as previously described [28].

### Seahorse mitochondrial stress assay

Seahorse XF24 analyzer and Wave desktop software v2.6.1 (Agilent Technologies, Inc., Santa Clara, CA, USA) were used for the assay of mitochondrial bioenergetic functions and data analysis, respectively. Mitochondria (6 μg protein in 50ul assay buffer) were seeded into each well, centrifuged at 2000 g for 20 min to attach the mitochondria to plate bottom, followed by addition of 450 μl warm assay buffer and treatments such as DEA-NONOate (NO donor) and H_2_O_2_. ADP (4 mM), Oligomycin (2.5 μg/ml), FCCP (4 μM), and Antimycin A (4 μM) were consecutively injected to reach different metabolic conditions as described previously [33].

### Flow cytometry assay

MACSQuant^®^ Analyzer 10 (Miltenyi Biotec, San Diego, CA, USA) and FLOWJo software v10.7.1 were used for flow cytometry assay and data analysis, respectively. The detailed methodology is given in S1 File and singlets were gates as described in S1 Fig.

### Statistics

Statistical analyses were carried out with Prism, v8.4.0 (Graphpad Software, La Jolla, CA). The ROUT module was used to identify outliers. Average values are given as mean ± SEM. One-way ANOVA with Dunnett’s post hoc analysis and paired t-test were applied as noted in figure legends.

## Results

### iNO study

**Fig 2:**
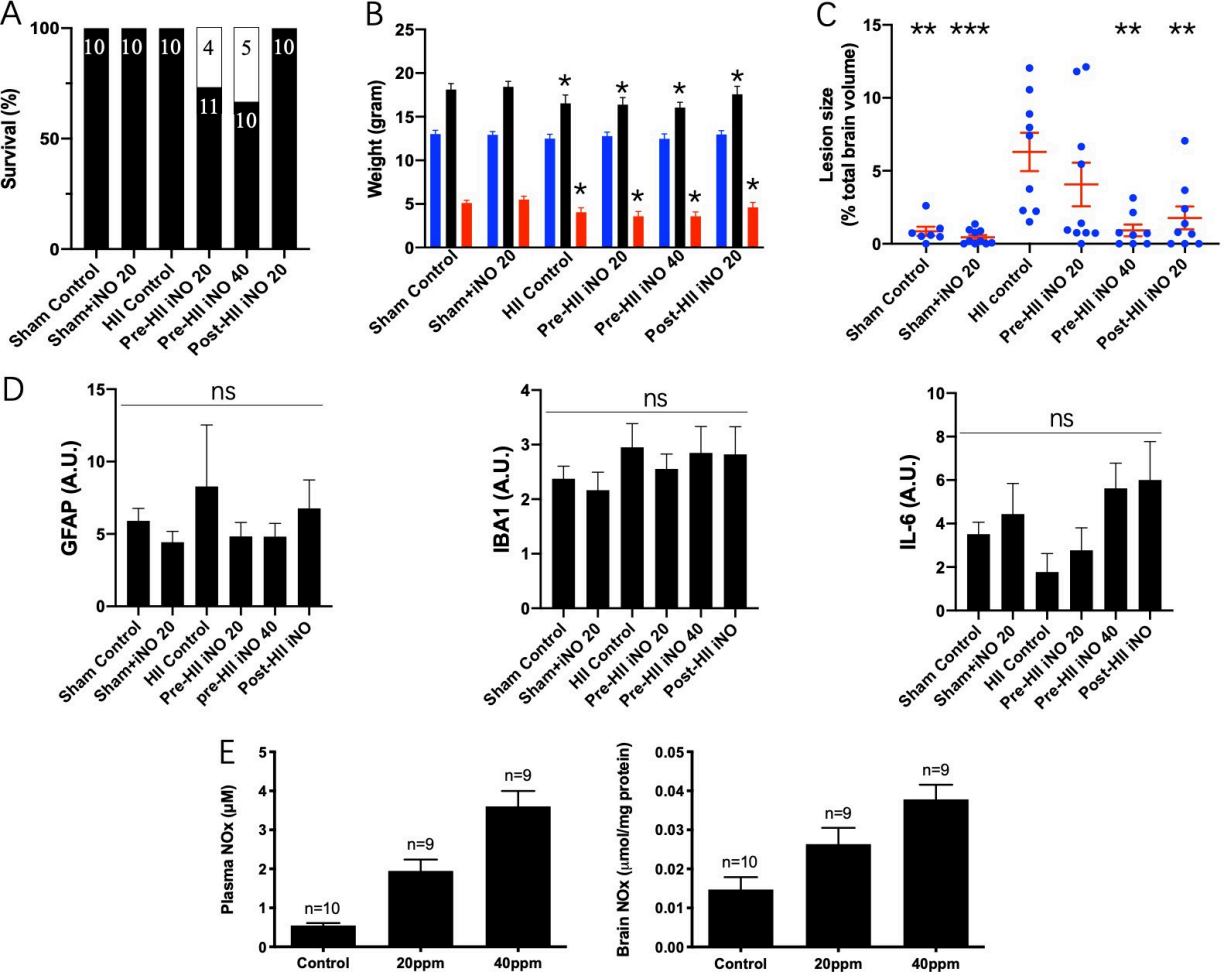
Effects of iNO on neonatal HII. **A)** Effects of iNO and HII on survival. pre-HII iNO at 20 and 40 ppm resulted in deaths. **B)** Effects of iNO and HII on weight (survived animals only). Blue represents baseline weight at P7, black represents weight at P10, red represents weight gained from P7 to P10 (*p<0.05 compared to Shams; One-way ANOVA). **C)** MRI analysis of infarct size at P10 (**p<0.01, *** p<0.001 vs HII control; One-way ANOVA). **D)** IHC analysis of brain slices at P10. Glial fibrillary acidic protein (GFAP) (left), ionized calcium binding adapter molecule 1 (IBA1) (middle), interleukin-6 (IL-6) (right). **E)** iNO increased NOx in plasma (left) and brain (right).

For the iNO study (Fig 1A), 27% and 37% of rat pups that received pre-HII iNO at 20 and 40 ppm, respectively, died before P10, while all other pups survived, including those that received post-HII iNO at 20 ppm (Fig 2A). These results suggest that iNO given before HII is toxic dose-dependently, while that given after (Post-HII iNO 20) or without (Sham+iNO 20) the HII is well tolerated. The baseline weight of pups on P7 was similar across all groups. However, HII resulted in less weight gain by P10, regardless of iNO treatment (*p<0.05 compared to Shams; survived animals only; One-way ANOVA; Fig 2B).

MRI analysis of brain infarct size at P10 confirmed the injury caused by HII (6.3±1.3% in HII control as compared to 0.9±0.3% in Sham control; Fig 2C). Despite the lower survival rates of pups in the pre-HII iNO 20 and 40 ppm groups, the lesion sizes of those that survived were 4.1±1.5% and 1.0±0.4%, respectively (p<0.01 vs HII control; one-way ANOVA). In addition, post-HII iNO at 20 ppm, which was well tolerated, significantly decreased the lesion size to 1.8±0. 8% (p<0.01 vs HII control; One-way ANOVA; Fig 2C), demonstrating the neuroprotective effects of post-HII iNO.

Nevertheless, IHC study of the same brains from MRI inspections failed to find any significant difference between groups with respect to prevalence of the inflammatory biomarkers GFAP, IBA1, and IL-6 (Fig 2D). These negative IHC results may represent inappropriate sample timing, region, biomarker, or the complexity of brain inflammatory responses.

Levels of NOx in both plasma and brain tissues increased in a dose-dependent manner following iNO (Figs 1B and 2E), confirming the successful administration of NO via inhalation. Although not further characterized, a major component of the NOx species was likely nitrite [34]. Based on evidence that nitrite itself is protective against ischemia-reperfusion injury [35], further experiments were conducted in which nitrite was administered instead of iNO.

### Nitrite study

**Fig 3:**
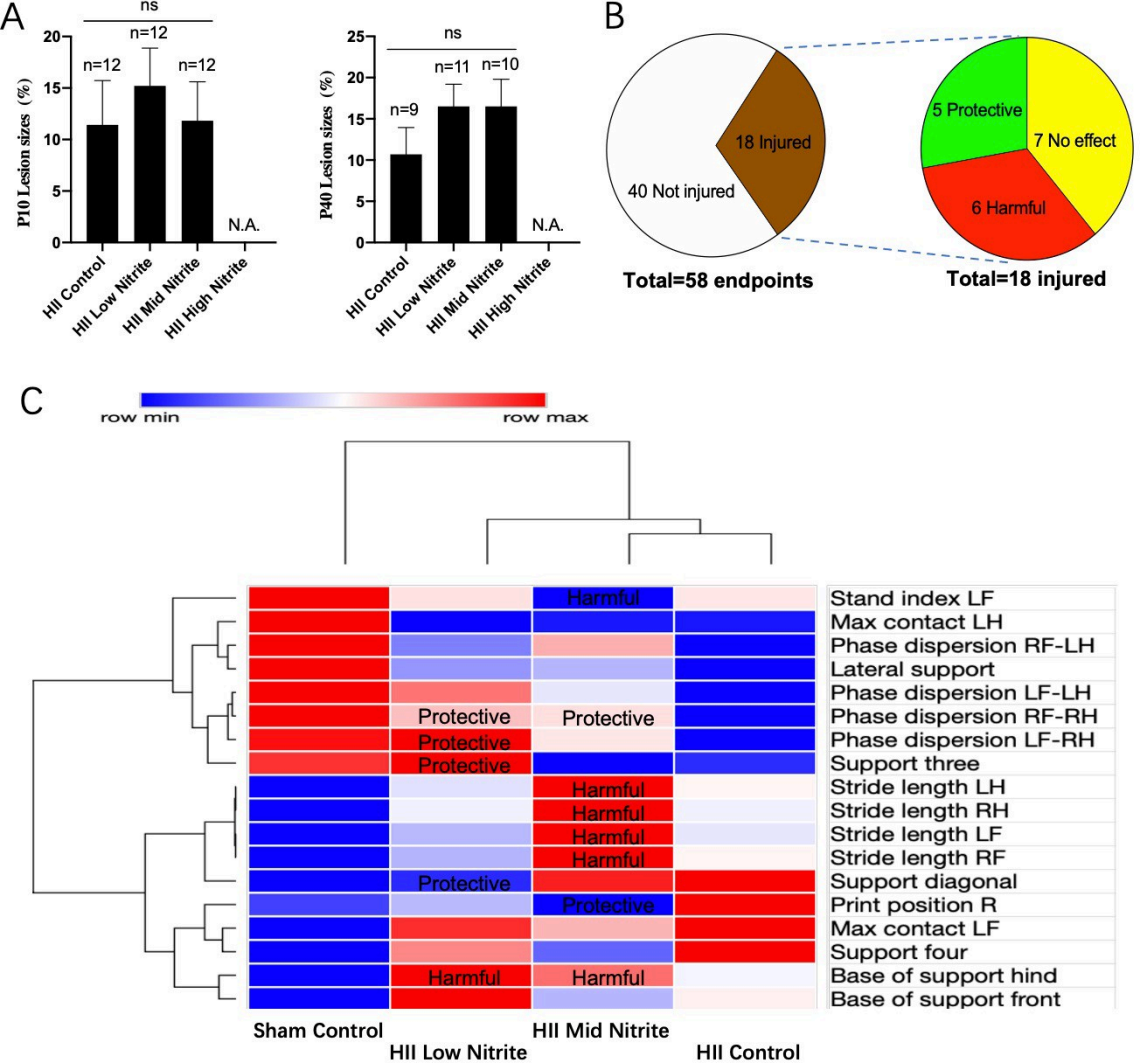
Effects of nitrite on neonatal HII. **A)** TTC-detected brain infarct size (as a percent of total brain volume) at P10 (left) and P40 (right). Nitrite at high dose resulted in death of all pups. **B, C)** Neurobehavioral effects of nitrite on HII at P40. **B)** In 58 gait endpoints, HII resulted in injury in 18, in which nitrite was protective in 5, harmful in 6, and not effective in 7. **C)** Heatmap of the quantitative gait analysis of the nitrite effects on HII injury. Group means were plotted with the minimum and maximum in each row assigned as blue and red, respectively, and analyzed by hierarchical clustering. Protective and harmful represent mitigation and deterioration of HII-induced alteration, respectively. RF: Right forepaw, LF: Left forepaw, RH: Right hindpaw, LH: Left hindpaw. Descriptions of gait endpoints and detailed data see S1–S3 Tables.

The effects of nitrite on HII were studied using the same HII model as above and following the protocol given in Fig 1C. Given that the conversion of nitrite into NO is favored under hypoxia [11, 12], nitrite was given right before the start of the hypoxic insult via i.p. injection. The high nitrite dose (16.5 μg/g) resulted in the death of all pups. All pups receiving middle (1.65 μg/g) and low (0.165 μg/g) doses of nitrite survived, however no decrease in brain lesion size was observed, as measured at P10 and P40 via TTC staining (Fig 3A). Neurobehavioral tests at P40 found that out of 58 gait endpoints, HII resulted in injury (alteration from Sham Control) as measured by 18 endpoints, in which nitrite was protective in 5, harmful in 6, and not effective in 7 (Fig 3B). Further assessment of these results (Fig 3C; statistics are given in S1 Table) showed that middle-dose nitrite was protective and harmful in 2 and 5 endpoints, respectively. In contrast, low-dose nitrite was protective and harmful in 4 and 1 endpoints, respectively. Together, these results demonstrated that although nitrite was toxic at high dose and not effective in decreasing brain lesion sizes at any dose, it may protect motor and coordination performance at low dose.

### EPR study

**Fig 4:**
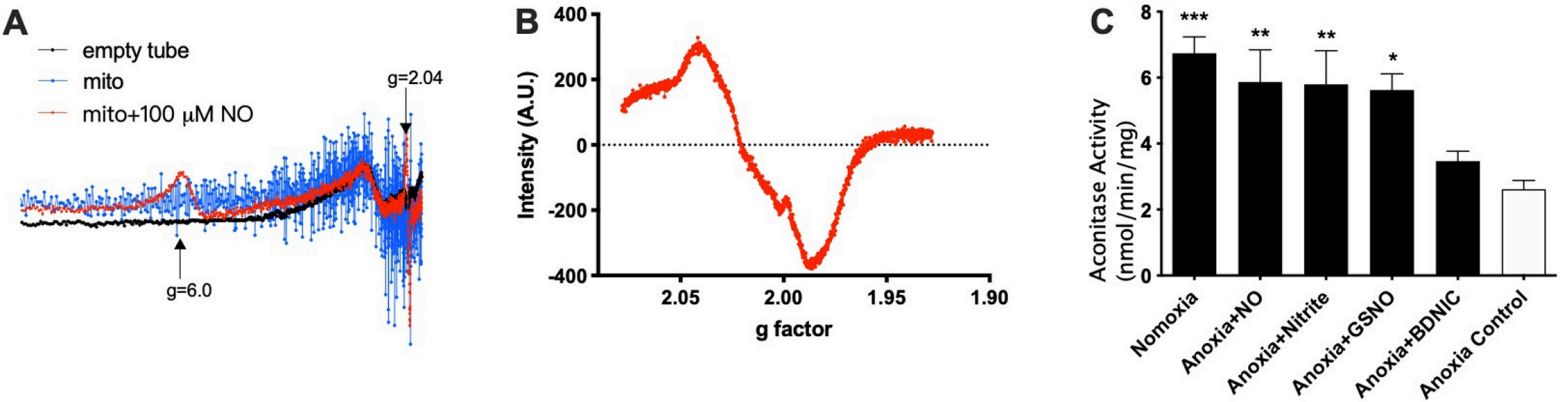
NO reacts with mitochondria and its antioxidant effects. **A, B)** EPR evidence for the generation of heme-NO complexes from binding of NO to mitochondria. **A)** Full EPR spectrum. **B)** Amplified **(A)** at an area with the g factor of around 2.0. **C)** Effects of NO and its metabolites (30 μM) on the aconitase activity, a marker of oxidative stress, of mitochondria subjected to anoxia. Mitochondria were isolated from brain cortex of fetal sheep, and challenged with anoxia (40 min anoxia+20 min reoxygenation). DEA-NONOate was used as source of NO (30 μM). * p<0.05, ** p<0.01, *** p<0.001 vs last bar in **C** (One-way ANOVA).

NO is known to react with mitochondria and generate various products including heme-NO, SNOs, which are proposed to be protective, and DNICs which are proposed to be harmful [14]. Taking advantage of EPR, which detects both heme-NO and DNICs, we investigated the reaction of NO with isolated brain mitochondria. As shown in Fig 4A and 4B, the presence of mitochondria did not alter the spectra observed from an empty EPR tube, whereas addition of NO to mitochondria resulted in two new peaks at g factors of ~6.0 and ~2.0, respectively. By comparison of the position and structure of the resonance lines with those in literature and those of standards (S2 Fig), these two peaks were identified as ferric-heme and heme-NO, respectively [36–39]. These results are consistent with previous findings that NO interacts with mitochondrial complex IV, cytochrome c oxidase (CcOX), by binding to the catalytic active heme *a*_3_ site [39–42].

### Aconitase activity study

Increasing evidence has suggested that aconitase, an iron sulfur protein that is a component of the Krebs cycle, is a target of NO in mitochondria [14]. Being very vulnerable to oxidative damage, the activity of aconitase has been used as an index of mitochondrial oxidative stress [43]. The anoxia-induced decrease in aconitase activity was attenuated by treatment of mitochondria with NO, nitrite, or GSNO (Fig 4C), suggesting these NO-related treatments had antioxidative effects.

### Mitochondrial oxygen consumption study

**Fig 5:**
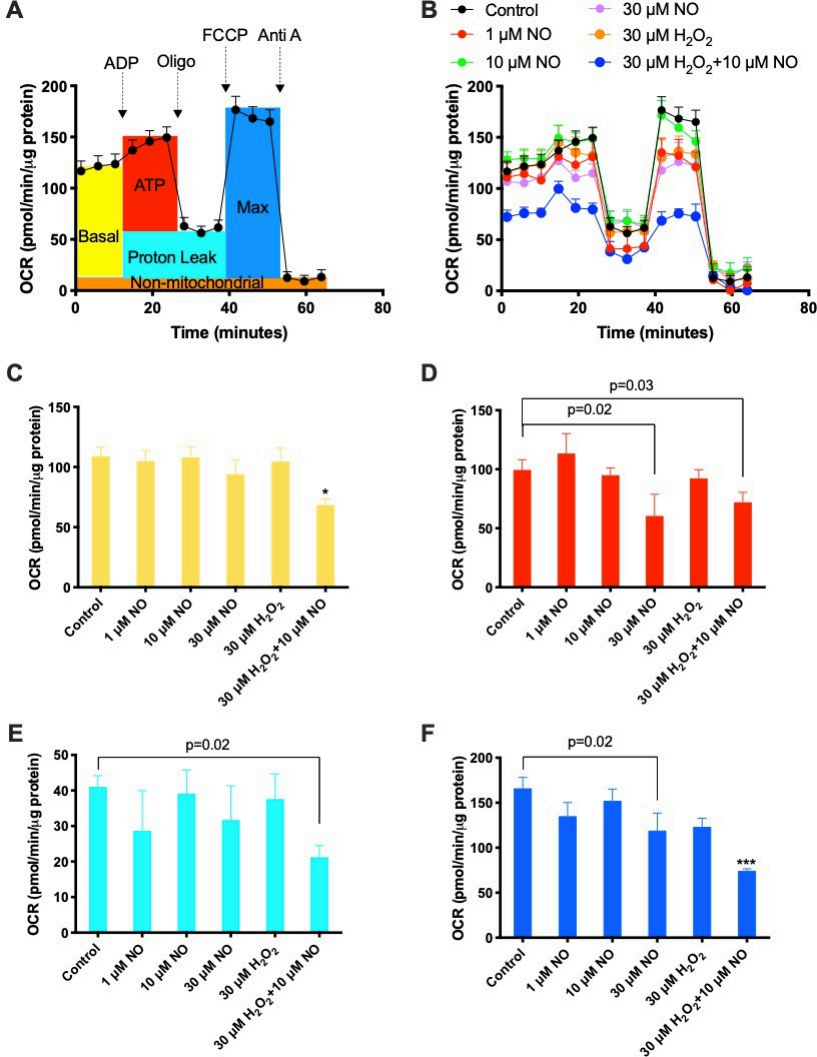
Effects of NO on mitochondrial oxygen respiration. n = 5. Seahorse measurements of the effects of NO on mitochondrial oxygen consumption rate (OCR). **A)** Example (control group) for the Seahorse assay of mitochondrial OCR. **B)** Representative OCR traces of mitochondria in the absence and presence of NO and/or H_2_O_2_. **C)** Basal respiration. **D)** ATP-linked respiration. **E)** Proton leak. **F)** Maximal respiration. * p<0.05, *** p<0.001 vs Control (One-way ANOVA). Given p value represents the result of paired t-test.

The interaction of NO and CcOX has been proposed to reversibly inhibit CcOX and thus oxidative phosphorylation (OXPHOS) [39–42]. To examine this possibility, we measured the effects of NO on mitochondrial oxygen consumption via Seahorse assay (Fig 5). While 30 μM NO did not affect basal respiration and proton leak, it did inhibit ATP-linked respiration and maximal respiration capacity, consistent with inhibition of CcOX and OXPHOS by NO. It is worth noting that although neither 10 μM NO nor 30 μM H_2_O_2_ had any effect on OXPHOS, the combination of these two resulted in a synergistic inhibitory effect (Fig 5B–5F).

### FACS study

Validation of the FACS methodology was obtained by noting the effects of CCCP, an uncoupler of oxidative phosphorylation, on mitochondrial membrane potential and size (S3 Fig). Further validation was obtained by demonstration of the effects of DMNQ, a redox-cycling ROS generator, and antimycin A, an inhibitor of complex III that leads to increased ROS production. Despite positive validation, 40 min anoxia plus 20 min reoxygenation failed to result in any detectable alteration in mitochondrial membrane potential, size, or ROS (S3A–S3C Fig). Parallel experiments with the same mitochondria demonstrated that aconitase activity was significantly decreased in response to anoxia (Fig 4C), indicating the above negative results cannot be explained by potential failure to induce anoxia. Although anoxia failed to significantly alter mitochondrial size, it is worth noting that NO tended to decrease the swelling (p = 0.0519; paired t-test) of mitochondria exposed to anoxia (S3B Fig). In addition, NO also tended to protect against the fall in mitochondrial membrane potential caused by CCCP (p = 0.1022; paired t-test; S3A Fig).

## Discussion

This study indicates that iNO, with cautions on timing and dose, has neuroprotective effects following HII in newborns, and suggests that the mechanism of protection is via its modulation of mitochondrial function. Our results demonstrated that post-HII iNO was well tolerated and provided strong protection against the HI-induced brain injury, while pre-HII iNO, in a dose-dependent manner, alleviated the HI-induced brain injury but also resulted in considerable decrease in survival. Pre-HII nitrite did not show any effect on HI-induced brain lesions. However, on neurobehavioral performance, nitrite was protective at low dose while detrimental at higher doses. Consistent with the neuroprotective effects found in vivo, NO decreased the oxidative stress of isolated brain mitochondria challenged with anoxia. In addition, NO suppressed the ATP-linked respiration and maximal respiration of mitochondria likely via its inhibitory reaction with the heme a_3_ of CcOX.

Although NO was first known as a toxic gas, its use as an inhaled therapy has established a strong clinical record of safety with the primary adverse effect of methemoglobinemia rarely observed. In the current studies, although iNO reduced brain lesion sizes when administered either before or after HII (Fig 2C), it was also found to decrease survival rates when administered prior to HII (Fig 2). The reason underlying this somewhat disparate finding may be that while iNO has neuroprotective effects, it is also detrimental to the overall cardiovascular response to HII. The Rice-Vannucci model employed in these experiments involves a whole-body response that includes a redistribution of cardiac output from peripheral tissues to critical organs such as the myocardium and brain. This aspect of the model mirrors clinical HII, where hypotension, hypotonia, acidemia, and damage in various peripheral organs is often observed [44]. Albeit subtle, iNO has been shown to have systemic vasodilatory effects, which are thought to be mediated by elevation of the circulating concentrations of metabolites of NO which have NO-like vasodilatory activity [45–47]. Furthermore, the most prominent of these metabolites, nitrite, is known to produce vasodilation more potently under hypoxic conditions [48]. Thus, it is possible that while iNO had protective effects in the brain, it may have impaired the cardiovascular response to systemic hypoxia such that overall mortality was increased. This possibility requires further examination in an animal model capable of instrumentation for measurement of cardiovascular function. It is also possible that pre-administration of iNO resulted in an elevation of NO metabolites that react with ROS generated during the hypoxic period. For example, NO produced from nitrite under hypoxia may react with superoxide to produce the highly toxic peroxynitrite anion. Likewise, due to the synergistic effects of NO and H_2_O_2_ on suppression of mitochondrial O_2_ consumption (Fig 5B–5F), pre-HII iNO may have resulted in more harmful inhibition of mitochondrial function. Nevertheless, because the decrease in survival was accompanied with decrease in brain lesion size (Fig 2A and 2C), it seems more likely that the lethal mechanism of pre-HII iNO lies in organs other than the brain. Further study is warranted, preferably in animal models large enough for more detailed monitoring of cardiovascular function, to evaluate the safety of iNO and elucidate the mechanisms underlying its harmful effects under hypoxia.

This study found that iNO was a more effective neuroprotectant than nitrite. Since the discovery of its NO-like bioactivity, nitrite has been intensively investigated as a surrogate of NO in various experimental models, particularly HII. There is compelling evidence that nitrite is protective, exerting its bioactivity via reduction to NO under hypoxic or acidic conditions [35]. These facts together with its wide safety margin, affordability, and relative ease of administration, make nitrite a promising possible alternative to iNO for the treatment of neonatal HII. However, it has also been reported that NO and nitrite have different metabolic fates in the mitochondria, and that these different metabolites might even lead to opposite effects on cytoprotection against HII [14], raising doubt about the use of nitrite as an NO surrogate. In the current study, iNO demonstrated stronger neuroprotective effects than nitrite, even in the pre-HII groups that had decreased survival. Although the neurobehavioral endpoints were not assessed in the iNO groups, iNO administration resulted in plasma nitrite concentrations of ~2 to 3 μM (Fig 2E) which is comparable to that expected to occur in the low dose nitrite group, and thus it is reasonable to speculate that iNO will have the same protective effects on neurobehavioral performance as those observed in the low dose nitrite group.

The current study supports the proposition of mitochondria-targeted strategy for combating HII. While the mechanisms underlying HII are not yet fully understood, the extent of mitochondrial dysfunction is known to correlate with overall neurological damage and long-term neurodisability [5, 6]. NO has been shown to modulate mitochondrial function, such as suppression of O_2_ consumption that may provide protection against injury, making it a candidate for mitochondria-targeted therapy [13–15]. Consistent with this idea, our study demonstrated in isolated brain mitochondria that NO inhibits OXPHOS and protects against anoxic and chemical challenges (Figs 4C and 5B and S3). In addition, both pre- and post- HII iNO decreased the brain infarct sizes in the neonatal rat model of HII (Fig 2C). Nevertheless, it is important to note that NO-mediated neuroprotection might not be limited to its inhibition of mitochondrial OXPHOS. For example, it has been reported that NO can activate the Keap1/Nrf-2 pathway and confer cellular protection against oxidative stress [49]. In addition, iNO has also been shown to selectively dilate arterioles in the ischemic penumbra and facilitate collateral blood flow in experimental stroke models [19, 20].

There are many possible ways in which NO can modulate mitochondrial function. For example, NO may cause S-nitrosylation of cysteine thiols in complex I and IV and inhibit OXPHOS, or inhibit NADPH oxidase and reduce superoxide production which may have downstream effects on mitochondrial function (see below) [13, 50–52]. Alternatively, NO may form DNICs possibly via releasing protein-bound iron from complexes such as iron-sulfur centers, resulting in toxic or protective effects [14, 53]. In addition, NO can cause glutathionylation of cysteine residues, N-nitrosation of amines, and nitration of tyrosine residues [54]. In this study, the role of CcOX (Complex IV) in NO-induced mitochondrial inhibition was supported by several lines of evidence. First, NO formed a ferric-heme and a heme-NO with mitochondria as identified by EPR, consistent with the previous findings that NO reacts with the heme *a*_3_ of CcOX to generate CcOX-NO_2_^-^ (*a*_3_^3+^Cu_B_^2+^NO_2_^-^; ferric-heme) and CcOX-NO (*a*_3_^2+^Cu_B_^+^NO; heme-NO) [39]. Second, NO decreased mitochondrial respiration rate in the presence of the uncoupler FCCP, which is likely associated with the inhibition of the CcOX in uncoupled mitochondria [15]. Lastly, with the presence of Complex I inhibitor rotenone in our assays, succinate, which provides FADH_2_ to Complex II through the Krebs cycle, stimulated OXPHOS only by Complex II-IV [33], excluding possible involvement of Complex I, a widely reported target of NO in mitochondria [13, 50, 51, 55].

One interesting finding of this study is the synergistic inhibition of NO and H_2_O_2_ on mitochondrial OXPHOS (Fig 5B–5F). It has been shown that H_2_O_2_ compromises mitochondrial function via inactivation of succinate dehydrogenase [56, 57]. Succinate dehydrogenase is not only a part of the Krebs cycle that oxidizes succinate, but also a subunit of Complex II of the electron transport chain [58]. In the assay buffer for mitochondrial assays, succinate was the only substrate for the OXPHOS. It is likely that H_2_O_2_ inactivated succinate dehydrogenase, and thus amplified the CcOX-targeted inhibitory effects of NO on OXPHOS via a two-target, two-hit model. In addition, H_2_O_2_ has been demonstrated to inactivate the Krebs cycle enzymes alpha-ketoglutarate dehydrogenase and aconitase [56, 57]. Furthermore, NO has also been shown to inhibit succinate dehydrogenase via interacting with its Fe-S cluster [59]. Whatever the mechanism might be, given that HI often induces production of H_2_O_2_, the synergy between NO and H_2_O_2_ may reinforce the inhibitory effects of NO on mitochondrial OXPHOS during HII.

The conclusions of that can be drawn from this study are limited by several factors. First, we were unable to assess the effects of iNO or nitrite on cardiopulmonary function of the neonatal rat pups, due to their small size. This is a chief limitation of the Rice-Vannucci model and, as noted above, the ability to monitor cardiovascular parameters such as blood pressure might have revealed the cause of increased fatalities in the pre-HII iNO groups. The neurobehavioral studies were also limited by the lack of a sham group that received only low-dose nitrite. As a result, it cannot be determined whether the mixed effects of nitrite on neurobehavioral endpoints in the HII-group would have occurred independent of the hypoxic-ischemic treatment. Finally, caution must be used in extrapolating the results of the mitochondrial function studies, which were conducted in mitochondrial from fetal sheep brain, to the in vivo studies which were conducted in neonatal rat pups. While this latter point may limit the conclusions that can be made about the mechanism of iNO effects on the rat brain, the use of sheep mitochondria may also be seen as a strength, given that the fetal sheep is a commonly-used preclinical animal model with a proven track record of translatability to clinical medicine.

## Conclusion

This study supports the post-insult application of low dose iNO for treatment of neonatal HII. In addition, NO protects mitochondria from insults of anoxia and reoxygenation, and suppresses mitochondrial oxygen respiration possibly via its inhibitory reaction with CcOX at heme *a*_3_ site, supporting NO as a mitochondria-targeted treatment for neonatal HII.

## Supporting information

S1 File Supplemental methods. (PDF)

S1 Table CatWalk parameter statistics following HII. (PDF)

S2 Table Full list of tested CatWalk parameters. (PDF)

S3 Table Definitions of the CatWalk XT parameters altered by HII. (PDF)

S1 Fig Gating of mitochondrial singlets on flow cytometry. **A**) Visualization of all events (mitochondria) in a dot plot. **B**) Singlets, as distinguished by plotting FSC-A versus FSC-H, were gated for further analysis. Doublets, which had less than half of the mean FSC-H/ FSC-A value of the majority of events, were excluded. **C**) Visualization of the remaining singlets in a dot plot.
(PDF)

S2 Fig EPR spectra of Ferric-Heme (A; MetHb) and Heme-NO (B; HbNO). MetHb powder was purchased from Sigma. HbNO was prepared by reaction of deoxy-Hb with N-14 nitrite under anoxia.
(PDF)

S3 Fig Flow cytometry measurements of effects of NO on challenged mitochondria. n = 5. Data were normalized to the first bar in each panel. **A)** Membrane potential, **B)** Size, and **C)** ROS. Mitochondria were isolated from brain cortex of fetal sheep, and challenged with anoxia (40 min anoxia+20 min reoxygenation), 15 μM free Ca^2+^, 50 μM CCCP, 20 μM DMNQ, or 10 μM AntiA. DEA NONOate was used as source of NO (30 μM). * p<0.05, ** p<0.01, *** p<0.001 vs first bar in each panel (One-way ANOVA). Given p value represents the result of paired t-test.
(PDF)
