# Supplementary material for: Neuroprotective role of nitric oxide inhalation and nitrite in a Neonatal Rat Model of Hypoxic-Ischemic Injury
Source: PLoS One. 2022 May 11;17(5):e0268282. doi: 10.1371/journal.pone.0268282 (PMC9094545; doi:10.1371/journal.pone.0268282)
Supplement: S1 File — (PDF) [file pone.0268282.s001.pdf]

Supplemental Methods:

Neurobehavioral tests

CatWalk XT® (Noldus Information Technology, Wageningen, Netherlands), a quantitative gait analysis system, was used to assess motor and coordination performance as previously described[1]. Animals were trained to walk across the glass platform towards the goal box for 4 days, allowing them to acclimate to the environment. On the fifth day (P40), the animals needed to complete 3 to 5 compliant runs in order to be considered complete. A run was only considered compliant if the duration was between 1 to 10 sec with <60% variation in their run speed. If an animal was unable to complete the required number of runs, the lowest score for the particular group was assigned. The experiments were carried out in a dark room. As the animals crossed an illuminated glass walkway, light was reflected off their paws producing a series of footprint images, which were recorded by a video camera located 75 cm underneath the walkway. Data from these recordings were collected and analyzed by the CatWalk XT software. A total of 58 endpoints were evaluated. A full list of all tested endpoints and a description of the endpoints that were found to be altered by HII are given in Supplemental Table 1 and 2, respectively. Several of these endpoints measured each individual limb or a combination of limbs as its own endpoint.

Mitochondria isolation

Mitochondria were isolated from the near-term (~140 gestation days; preterm at ~130, term at ~150 days) fetal sheep brain cortex using a modified Percoll density gradient centrifugation methodology[2][3]. Briefly, brain tissue was rinsed free of blood and homogenized in ice-cold isolation buffer (1 gram in 8 ml) in glass douncers (10 loose strokes followed by 15 tight strokes). The homogenate was centrifuged at 500 g for 5 min to remove unlysed cells and tissue debris. The supernatant was further centrifuged at 14,000 g for 10 min. The resulting supernatant was aspirated off, and the pellet, containing mitochondria, was resuspended in 1 ml of 12% Percoll. The suspension was carefully layerd on top of 5 ml of 21%

Percoll and centrifuged at 18,000 g for 15 min to further isolate mitochondria from other cellular substances. Then, the Percoll was removed by centrifugation as follows. First, the 3.5 ml top portion was aspirated off and the 6 ml isolation buffer was added to the remaining 2.5 ml bottom portion followed by centrifugation at 18,000 g for 5 min. Second, 7.5 ml was aspirated from the top, and 7.5 ml of isolation buffer was added to the 1 ml bottom portion followed by centrifugation at 14,000 g for 5 min. The resulting solid pellets, the mitochondria, were resuspended in 0.5 ml assay buffer for assays within 2 hours. The isolation buffer contained (in mM; pH 7.2): mannitol 210, sucrose 70, HEPES 5, EGTA 1 and 0.5% (w/v) BSA (fatty acid free). Percoll solution (100%) was prepared by dissolving above compounds in Percoll® (Sigma-Aldrich, St. Louis, MO, USA), and was diluted with isolation buffer to 12 and 21%. Assay buffer contains (in mM; pH 7.2): mannitol 220, sucrose 70, KH_2PO_4 10, $MgCl_2$ 5, HEPES 2, EGTA 1, succinate 10, rotenone (2 μM), and 0.2% (w/v) BSA (fatty acid free). An Optima XPN ultracentrifuge (Beckman Coulter, Brea, CA, USA) was used for centrifugations.

Flow cytometry assay

MACSQuant® Analyzer 10 (Miltenyi Biotec, San Diego, CA, USA) and FLOWJo software v10.7.1 were used for flow cytometry assay and data analysis, respectively. Mitochondria (0.4 μg/μl) in assay buffer containing 4 mM ADP were stained with fluorescence probes (Thermo Fisher Scientific, MA, USA) and treated with anoxia, DEANONOate (NO donor), $CaCl_2$, CCCP, DMNQ, and/or Antimycin A at 37 ^oC for 40 min. Samples were injected at "medium" setting of flow rate, and singlets were gated (Supplemental Figure 1) by plotting FSC-A versus FSC-H (linear mode; 490 v) for fluorescence analysis. Double staining of NAO (100 nM; 10-nonyl acridine orange; Ex=488 nm/Em=525 nm; B1 channel, 400 v) and DILC1(5) (10 nM; Ex=638 nm/Em=658 nm; R1 channel, 450 v) were used to detect the membrane potential of mitochondria as previously described (35). Single staining of MitoSOX™ Red (5 μM; Ex=510 nm/Em=580 nm; B2 channel, 400 v) was used to detect the ROS (superoxide) level in mitochondria. The mitochondria swelling was detected by an increase in FSC.

References:

1. Knox-Concepcion KR, Figueroa JD, Hartman RE, Li Y, Zhang L. Repression of the Glucocorticoid Receptor Increases Hypoxic-Ischemic Brain Injury in the Male Neonatal Rat. Int J Mol Sci. 2019;20. doi:10.3390/ijms20143493

2. Sims NR, Anderson MF. Isolation of mitochondria from rat brain using Percoll density gradient centrifugation. Nat Protoc. 2008;3: 1228–1239. doi:10.1038/nprot.2008.105

3. Chinopoulos C, Zhang SF, Thomas B, Ten V, Starkov AA. Isolation and functional assessment of mitochondria from small amounts of mouse brain tissue. Methods Mol Biol. 2011;793: 311–324. doi:10.1007/978-1-61779-328-8_20
