# Supplementary material for: Neuroprotective role of nitric oxide inhalation and nitrite in a Neonatal Rat Model of Hypoxic-Ischemic Injury
Source: PLoS One. 2022 May 11;17(5):e0268282. doi: 10.1371/journal.pone.0268282 (PMC9094545; doi:10.1371/journal.pone.0268282)
Supplement: S1 Table — (PDF) [file pone.0268282.s002.pdf]

S1 Table. CatWalk parameter statistics following HII

Parameters	Sham	Control	0.165	1.65	Effect of Nitrite
Max contact LH	39.15±1.73	30.16±2.02	29.71±1.73	30.17±1.66	C
Max contact LF	41.92±0.63	46.91±1.19	46.46±0.83	45.13±1.45	C
Stand index LF	-6.86±0.27	-8.57±0.35	-8.53±0.49	-10.6±0.55	B
Stride length LH	3.51±0.06	3.79±0.06	3.75±0.06	4.05±0.08	B
Stride length LF	3.56±0.05	3.81±0.07	3.76±0.06	4.11±0.06	B
Stride length RH	3.45±0.06	3.75±0.07	3.75±0.06	4.08±0.07	B
Stride length RF	3.53±0.05	3.82±0.06	3.73±0.07	4.09±0.07	B
Print position R	-0.057±0.034	0.142±0.059	-0.003±0.047	-0.087±0.041	A
Phase dispersion LF-LH	64.15±0.69	59.89±1.18	63.16±1.02	61.83±1.07	C
Phase dispersion RF-RH	65.05±0.7	58.24±0.96	62.47±0.97	62.05±0.73	A
Phase dispersion LF-RH *	14.85±0.72	8.38±1.22	15.06±1.15	12.02±0.9	A
Phase dispersion RF-LH	18.31±1.98	10.35±1.11	12.38±0.83	15.54±2.23	C
Lateral support	12.18±0.83	7.22±1.01	8.69±1.01	9±0.86	C
Support four	0.86±0.21	2.92±0.74	2.37±0.63	1.27±0.51	C
Support three	38.17±2.04	29.95±2.09	39.18±2.27	29.06±2.52	A
Support diagonal	46.19±1.79	58.26±2.38	47.21±2.54	57.49±2.33	A
Base of support hind	2.94±0.04	3.18±0.07	3.44±0.07	3.33±0.08	B
Base of support front	2.01±0.04	2.2±0.08	2.37±0.07	2.14±0.06	C

A: Protective, B: Harmful, C: No effect

If there was benefit or harm for either dose of nitrite, the parameter was categorized as such.

*Phase dispersion LF-RH had significant outliers thus data is presented with outliers removed and Kruskal-Wallis test was used for data analysis prior to removal of outliers to identify significance.

RF- Right Forepaw, LF – Left Forepaw, RH – Right Hindpaw, LH – Left Hindpaw
