# Supplementary material for: Neuroprotective role of nitric oxide inhalation and nitrite in a Neonatal Rat Model of Hypoxic-Ischemic Injury
Source: PLoS One. 2022 May 11;17(5):e0268282. doi: 10.1371/journal.pone.0268282 (PMC9094545; doi:10.1371/journal.pone.0268282)
Supplement: S2 Table — (PDF) [file pone.0268282.s003.pdf]

S2 Table. Full list of tested CatWalk parameters.

Parameter #	Parameter name
1	Base of Support Front
2	Base of Support Hind
3	Duty Cycle LF
4	Duty Cycle LH
5	Duty Cycle RF
6	Duty Cycle RH
7	Intensity LF
8	Intensity LH
9	Intensity RF
10	Intensity RH
11	Lateral Support
12	Max Contact Area LF
13	Max Contact Area LH
14	Max Contact Area RF
15	Max Contact Area RH
16	Max Contact at LF
17	Max Contact at LH
18	Max Contact at RF
19	Max Contact at RH
20	Paw Area LF
21	Paw Area LH
22	Paw Area RF
23	Paw Area RH
24	Phase Dispersion LF-LH
25	Phase Dispersion LF-RF
26	Phase Dispersion LF-RH
27	Phase Dispersion LH-RH
28	Phase Dispersion RF-LH
29	Phase Dispersion RF-RH
30	Print Position LF
31	Print Position RT
32	Regularity Index
33	Run Duration
34	Stand Duration LF
35	Stand Duration LH
36	Stand Duration RF
37	Stand Duration RH

38	Stand Index LF
39	Stand Index LH
40	Stand Index RF
41	Stand Index RH
42	Stride Length LF
43	Stride Length LH
44	Stride Length RF
45	Stride Length RH
46	Support Diagonal
47	Support Four
48	Support Girdle
49	Support Single
50	Support Three
51	Swing Duration LF
52	Swing Duration LH
53	Swing Duration RF
54	Swing Duration RH
55	Swing Speed LF
56	Swing Speed LH
57	Swing Speed RF
58	Swing Speed RH
