# Supplementary material for: Neuroprotective role of nitric oxide inhalation and nitrite in a Neonatal Rat Model of Hypoxic-Ischemic Injury
Source: PLoS One. 2022 May 11;17(5):e0268282. doi: 10.1371/journal.pone.0268282 (PMC9094545; doi:10.1371/journal.pone.0268282)
Supplement: S3 Table — (PDF) [file pone.0268282.s004.pdf]

S3 Table. Definitions of the CatWalk XT parameters altered by HII.

Parameter (unit)	Definition
Run duration (s)	Time duration of entire run
Regularity Index (%)	Four times the number of step sequences over the total number of paw steps
Duty cycle (% time)	Percentage of time the paw accounts for the total step cycle
Print position (mm)	Distance from forepaw position to consecutive hindpaw position
Base of support (mm)	Distance between girdle paw pairs
Support (%)	Percentage of time standing on set number of paws
Phase dispersion (%)	Percentage of time the target paw takes to step in relation to the step cycle of the anchor paw
Stand duration (s)	Time duration of the paw in contact with the floor during a step cycle
Stand index (index ratio)	Index is an indication of the speed the paw is lifted from the floor
Swing duration (s)	Time duration between two consecutive steps of the paw during a step cycle
Swing speed (m/s)	Stride length over the swing duration
Stride length (mm)	Distance the paw travels from one step to the next
Paw area (mm^3)	Size of the paw in contact with the floor
Max contact (%)	Percentage of time the paw takes to get to maximum contact with the floor over the total stand duration
Max contact area (mm^3)	Size of the paw at maximum contact with the floor
Intensity (0-255 a.u.)	Pressure of paw in contact with the floor
