# Supplementary figures and images for: Neuroprotective role of nitric oxide inhalation and nitrite in a Neonatal Rat Model of Hypoxic-Ischemic Injury
Source: PLoS One. 2022 May 11;17(5):e0268282. doi: 10.1371/journal.pone.0268282 (PMC9094545; doi:10.1371/journal.pone.0268282)

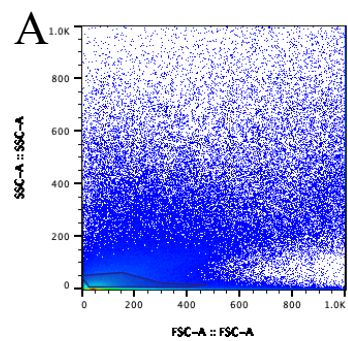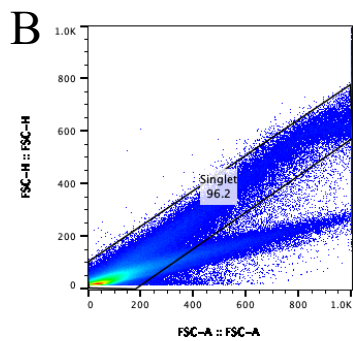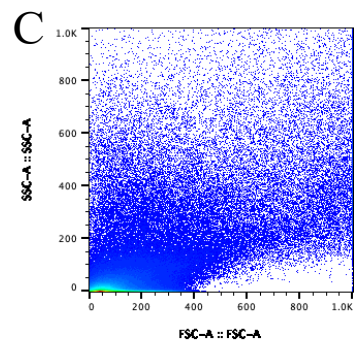

Supplement: S1 Fig — A) Visualization of all events (mitochondria) in a dot plot. B) Singlets, as distinguished by plotting FSC-A versus FSC-H, were gated for further analysis. Doublets, which had less than half of the mean FSC-H/ FSC-A value of the majority of events, were excluded. C) Visualization of the remaining singlets in a dot plot. (PDF) [file pone.0268282.s005.pdf]

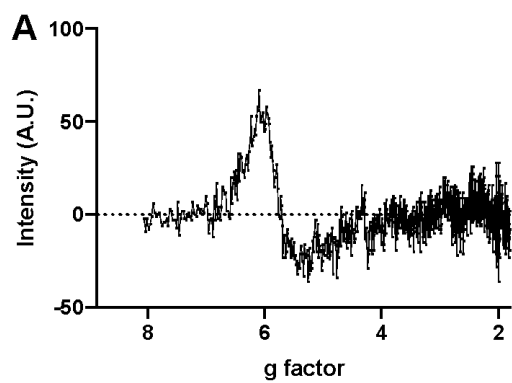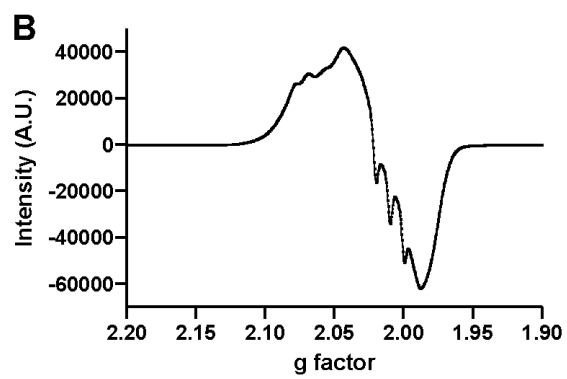

Supplement: S2 Fig — MetHb powder was purchased from Sigma. HbNO was prepared by reaction of deoxy-Hb with N-14 nitrite under anoxia. (PDF) [file pone.0268282.s006.pdf]

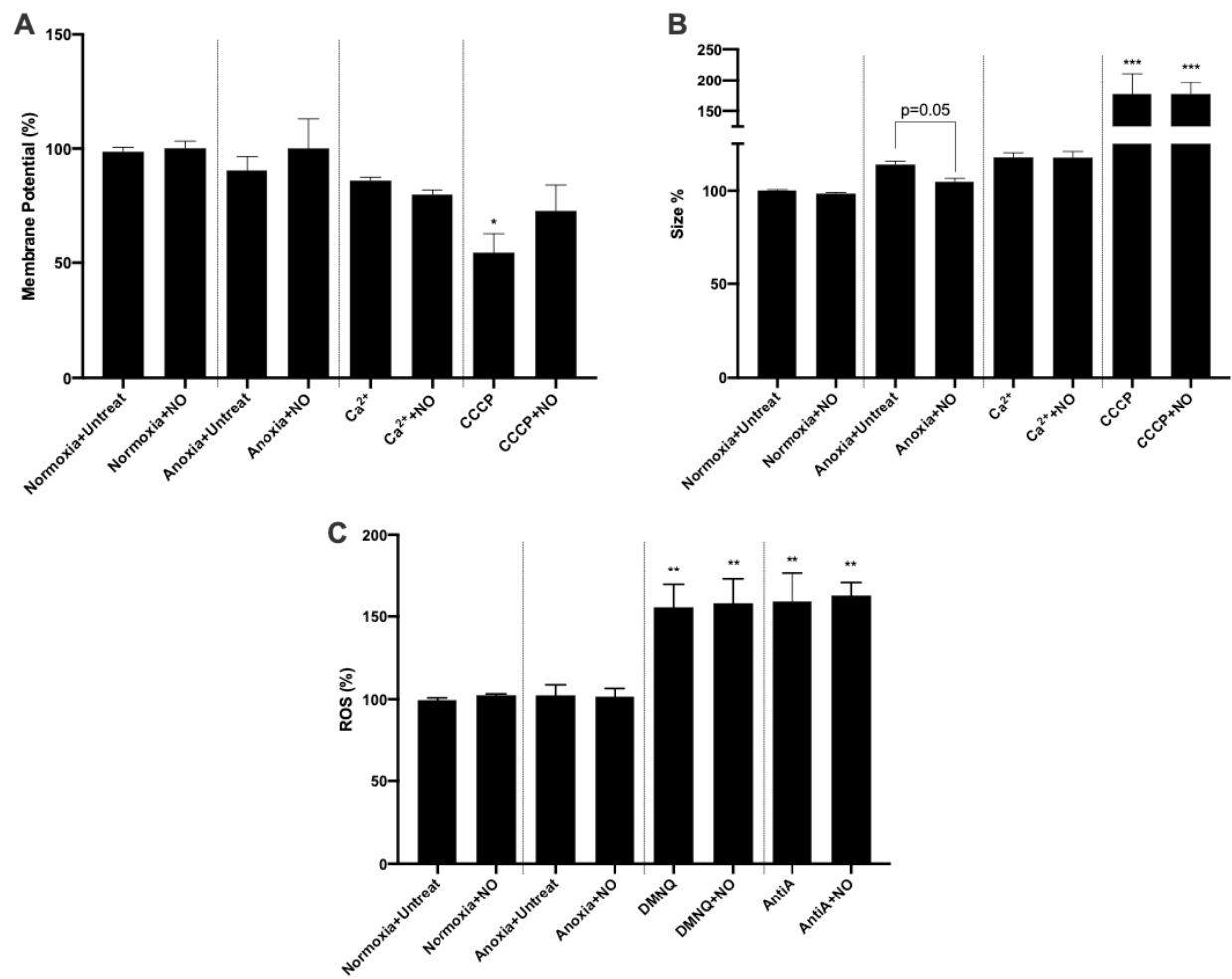

Supplement: S3 Fig — n = 5. Data were normalized to the first bar in each panel. A) Membrane potential, B) Size, and C) ROS. Mitochondria were isolated from brain cortex of fetal sheep, and challenged with anoxia (40 min anoxia+20 min reoxygenation), 15 μM free Ca2+, 50 μM CCCP, 20 μM DMNQ, or 10 μM AntiA. DEA NONOate was used as source of NO (30 μM). * p<0.05, ** p<0.01, *** p<0.001 vs first bar in each panel (One-way ANOVA). Given p value represents the result of paired t-test. (PDF) [file pone.0268282.s007.pdf]
